# Vitamin D receptor gene polymorphisms, bone mineral density and fractures in postmenopausal women with osteoporosis

**DOI:** 10.1007/s11033-012-2072-3

**Published:** 2012-10-17

**Authors:** Wanda Horst-Sikorska, Joanna Dytfeld, Anna Wawrzyniak, Michalina Marcinkowska, Michał Michalak, Edward Franek, Luiza Napiórkowska, Natalia Drwęska, Ryszard Słomski

**Affiliations:** 1 Department of Family Medicine, Poznań University of Medical Sciences, Przybyszewskiego Street 49, 60-355 Poznań, Poland; 2 Department of Computer Science and Statistics, Poznań University of Medical Sciences, Dąbrowskiego Street 79, 60-529 Poznań, Poland; 3 Department of Internal Medicine, Endocrinology and Diabetology, Central Clinical Hospital MSWiA, Wołoska Street 137, 02-507 Warszawa, Poland; 4 Department of Human Epigenetics, Medical Research Center, Polish Academy of Sciences, Pawinskiego Street 5, 02-106 Warsaw, Poland; 5 Department of Biochemistry and Biotechnology, Poznan University of Life Sciences, Wołyńska Street 35, 60-697 Poznań, Poland

**Keywords:** Osteoporosis, Osteoporotic fractures, Postmenopausal, Vitamin D receptor, VDR gene polymorphism

## Abstract

The goal of the study was to investigate the possibility of an association between polymorphisms and single alleles of *Bsm*I*, Apa*I*, Taq*I of the vitamin D receptor (VDR) gene with bone mineral density (BMD) and prevalence of vertebral/non-vertebral fractures in a group of postmenopausal Polish women with osteoporosis. The study group comprised of 501 postmenopausal females with osteoporosis (mean age 66.4 ± 8.9), who were diagnosed on the basis of either the WHO criteria or self-reported history of low-energy fractures. The three polymorphisms were determined by PCR (polymerase chain reaction) and RFLP (restriction fragment length polymorphism). BMD at the lumbar spine and femoral neck was assessed by dual energy X-ray absorptiometry (DXA). 285 fractures were reported in the whole group (168 vertebral and 117 non-vertebral). Incidence of non-vertebral fractures was significantly higher in the carriers of single alleles *a* of *Apa*I, *b* of *Bsm*I and *T* of *Taq*I VDR gene polymorphisms (*p* = 0.021, 0.032, 0.020, respectively). No significant associations between allelic variants of the studied polymorphisms and BMD or fracture incidence were found. (1).The presence of single alleles *a,b* and *T* of *Apa*I*, Bsm*I*, Taq*I VDR gene polymorphisms respectively, might serve as an indicator of non-vertebral fractures. (2). Lack of association between the VDR gene polymorphisms and BMD suggests that VDR contributes to low-energy fractures also through other ways.

## Introduction

Osteoporosis is a skeletal disease characterized by mechanical weakness and decreased bone mineral density (BMD), with susceptibility to fractures with minimal or no trauma. According to the World Health Organization (WHO) criteria, a densitometric evaluation—T score < −2.5 SD in the hip or lumbar spine—is required in order to diagnose osteoporosis. It is estimated that approximately 50 % women over the age of 80 will be diagnosed with osteoporosis (taking into account the annual bone loss after menopause), but only some percentage of them will suffer from the most important clinical manifestation of this disease—fractures [1, 2].

Predominant number of reported low-energy fractures occurs in women who do not meet the densitometric WHO criteria [2], leading to the conclusion that not only BMD itself but other factors, including environmental ones, have impact on the fracture risk as well. Several risk factors have been included into the FRAX™ calculator, that allows to calculate the 10 year fracture risk, even without the BMD data [3]. In the light of existing evidence, a very important role is attributed to genetic factors, not only because of their recognizable influence of BMD (estimated heritability ranges from 60–85 % for both hip and spine BMD and about 50 % for fracture, based on twin studies [4]) but also that fact that several agents contributing to bone microarchitecture (body mass index (BMI), age at menarche and menopause) are strongly genetically affected.

Our knowledge about the mechanisms by which vitamin D regulates bone metabolism, as well as its various extraskeletal activities, is steadily increasing. It seems there is no biological activity vitamin D does not participate in; it applies both to blood pressure regulation and carcinogenesis [5].

Over the last 20 years numerous candidate-genes for osteoporosis have been examined, i.e. parathormone (PTH) receptor, estrogen receptor, calcitonin receptor, IL-1 receptor antagonist, type I collagen, osteocalcin, peroxisome proliferators-activated receptor [6, 7]. Because vitamin D is one of the most important compounds whose effect on bone metabolism has been proven, a lot of interest has been expressed in its molecular mechanisms of action, particularly, in the vitamin D receptor (VDR). Over the years, it has become the most widely examined candidate-gene in osteoporosis. Despite the fact that the association between the VDR genotype and fracture risk has been investigated for years, there are still new, non-genomic interactions regulating genotype-phenotype relation.

VDR is a nuclear transcription factor, that mediates the action of 1,25(OH)_2_D3, thus affecting calcium absorption, bone remodeling and mineralization rate [8]. It is located on the long arm of 12. chromosome (3q11 locus), consists of 11 exons, 2–9 of which are actively transcribed. Since the pioneering work of Morrison in 1994, who demonstrated the association of the 3′ region VDR gene polymorphisms (*Bsm*I*, Apa*I*, Taq*I were identified) with BMD [9], numerous subsequent papers have been published on that topic. Their results have been inconclusive; still there is no clear answer whether specific VDR genotype is associated with lower BMD and higher fracture risk.

## Aim

The aim of the study was to ascertain whether there is an association between polymorphisms and single alleles of *Bsm*I*, Apa*I*, Taq*I of VDR gene with BMD and the prevalence of vertebral/non-vertebral fractures in a group of postmenopausal women with osteoporosis.

## Materials and methods

The study group consisted of 501 postmenopausal Caucasian women, aged between 47 and 88 years (mean 66.4 ± 8.9 years), with diagnosed osteoporosis. The diagnosis was established either on the basis of previous low-energy fractures or WHO densitometric criteria (T score < −2.5 SD). Low-energy fracture was self-reported and classified as such in cases when the following occurred:

1. Minor trauma or fall from standing height or less, preceding the fracture
2. Fracture at the age of 50 or later

They were all consecutive, non-institutionalized patients of the Endocrinology Outpatient Clinic, University Hospital No. 2, Poznań, and the Osteoporosis Center, Central Hospital of the Ministry of Interior and Administration, Warsaw. All participants gave their written informed consent to participate in the study.

Medical history of all participants, particularly regarding previous osteoporotic fractures, was taken. Subsequently, the group was further divided according to fracture location. History and clinical examination allowed to exclude patients with co-morbidities or medications as potential causes of secondary osteoporosis (i.e. hyperthyroidism, hyperparathyroidism, Cushing’s disease, kidney disease, rheumatoid arthritis, malabsorption syndromes). Dual X-ray absorptiometry (DXA) of the lumbar spine (L1-L4) and the hip was performed by means of Lunar DPX-L (Lunar Inc., Madison, WI, USA). The apparatus was calibrated daily. Measurements were performed using standard procedure according to the manufacturer’s recommendations.

## Genotyping

### *Bsm*I*, Apa*I, *Taq*I VDR gene polymorphisms

DNA was isolated from peripheral blood leukocytes by the guanidinium isothiocyanate method. The polymerase chain reaction (PCR) was carried out in 20 μl with 200 ng genomic DNA, 50 mM KCl, 10 mM Tris–HCl (pH 8.3), 1.5 mM MgCl2, 0.25 mM dNTP, 7.5 pmol of each primer, and 0.5 units of *Taq* polymerase (Sigma). The reaction was conducted as follows:

Initial denaturation at 94 °C for 4 min; denaturation at 94 °C or 40 s; primer annealing for 40 s; elongation at 72 °C for 100 s; final incubation at 72 °C for 180 s. For BsmI polymorphism, 837 bp fragment was amplified at annealing temperature of 55 °C for 35 cycles using primers F GGC AAC CAA GAC TAC AAG TAC C and R TCT TCT CAC CTC TAA CCA GCG [10]. For ApaI and* Taq*I polymorphisms of the *VDR* gene, 745 bp fragment was amplified at annealing temperature of 64 °C for 35 cycles using primers F CAG AGC ATG GAC AGG GAG CAA and R GCA ACT CCT CAT GGC TGA GGT CTC [11]. The PCR product was then subjected to restriction fragment length polymorphism (RFLP) analysis using the following restriction enzymes: *Taq*I, restrictase *Taq*I (Fermentas); ApaI, restrictase *Bsp*120I (Fermentas); BsmI, restrictase *Mva*1269I (Fermentas). All analyses were carried out according to the manufacturer’s recommendations, and the products of hydrolysis were separated on 1.5 % agarose gel and visualized with ethidium bromide.

Number of single alleles was calculated as a sum of a double number of homozygotes (dominant or recessive) and a single number of heterozygotes of each studied allele.

## Statistical analysis

All data are expressed as the mean ± SD, unless otherwise stated. The analyzed data were expressed on an interval and nominal scale. To compare the two groups, Student’s *t* test was performed or, in the absence of compliance with the required assumptions (normality and homogeneity of variance), the Mann–Whitney test. The Chi-square test was used to analyze nominal data. When comparing more than two groups simultaneously, univariate analysis of variance with Tukey post hoc test was done. In the case of non-compliance with normal distribution or lack of homogeneity of variance, Kruskal–Wallis test with Dunn’s post hoc tests were performed. All tests were analyzed at significance level of α = 0.05. Statistical analysis was done using Statistica 8.0 software (Stat Soft Inc, Tulsa, USA).

The study protocol was approved by Bioethical Committee at Poznan University of Medical Sciences, Poland and Medical University of Warsaw, Poland.

## Results

The baseline characteristics of the 501 studied women are presented in Table 1.

**Table 1:** Baseline characteristics of the study group

	Whole group (±SD)	Non-fracture group (±SD)	Fracture group (±SD)	p*
*n*	501	216	285	
Age (years)	66.4 ± 8.9	63.5 ± 9.1	68.5 ± 8.2	*p* < 0.0001
Body weight (kg)	62.7 ± 1–0.6	62.0 ± 10.4	63.1 ± 10.7	*p* = 0.6413
Height (cm)	156.7 ± 6,1	159.0 ± 5.7	155.0 ± 5.8	*p* < 0.0001
BMI (kg/m^2^)	25.4 ± 4.1	24.4 ± 3.8	26.2 ± 4.2	*p* = 0.9682
DEXA parameters				
L1–L4 BMD (g/cm^2^)	0.842 ± 0.148	0.889 ± 0.152	0.808 ± 0.135	*p* = 0.6413
L1–L4 T score SD	−2.7 ± 0.91	−2.47 ± 1.30	−3.04 ± 1.93	*p* < 0.0001
L1–L4 Z score SD	−1.3 ± 1.65	−1.01 ± 1.08	−1.24 ± 1.09	*p* = 0.0024
Hip BMD (g/cm^2^)	0.695 ± 0.088	0.717 ± 0.097	0.684 ± 0.080	*p* = 0.0063
Hip T score SD	−2.33 ± 0.59	−2.26 ± 0.80	−2.34 ± 0.74	*p* = 0.3916
Hip Z score SD	−0.72 ± 0.78	−0.74 ± 0.77	−0.68 ± 0.79	*p* = 0.4557

* Fracture versus non-fracture group

The mean age of the whole group was 66.4 ± 8.9 years. Females with the fracture history (regardless of its location) were significantly older, shorter, had lower lumbar T score and Z score, as well as hip BMD.

**Fig. 1:**
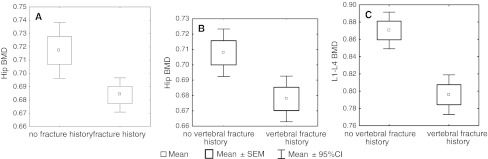
BMD in patients with and without fractures, depending on location

Among the subjects, 285 (56.8 %) underwent low-energy fracture, which was either of vertebral (168 cases) or non-vertebral (117 cases) location. In patients with previous fractures (regardless of its location), hip BMD significantly lower when compared to females without fracture (0.684 ± 0.080 vs. 0.717 ± 0.097 g/cm^2^, *p* = 0.006) (Fig. 1a). Among patients with vertebral fractures, both L1–L4 (0.796 ± 0.124 vs. 0.870 ± 0.154 g/cm^2^) and hip BMD (0.708 ± 0.094 vs. 0.694 ± 0.089 g/cm^2^) were significantly lower when compared to subjects without fracture (Fig. 1B, C). No relation between L1–L4 BMD and the investigated fracture incidence was found.

## BMD and VDR genotype

Association analysis of the studied polymorphisms is presented in Tables 2 and 3.

**Table 2:** BMD, T score and Z score in individuals with the BsmI, ApaI and* Taq*I—genotypes

DXA parameters^±^	BsmI genotype			*p*	ApaI genotype			*p*	*Taq*I genotype			*p*
	BB (*n* = 82)	Bb (*n* = 225)	bb (*n* = 193)		AA (*n* = 107)	Aa (*n* = 295)	aa (*n* = 135)		TT (*n* = 199)	Tt (*n* = 218)	tt (*n* = 84)	
BMD L1–L4 (g/cm^2^)	0.862 ± 0.15	0.845 ± 0.15	0.835 ± 0.14	ns	0.847 ± 0.14	0.836 ± 0.15	0.853 ± 0.14	ns	0.830 ± 0.14	0.846 ± 0.15	0.868 ± 0.15	ns
T score SD L1–L4	−2.68 ± 1.32	−2.63 ± 1.28	−2.91 ± 2.13	ns	−2.69 ± 1.30	−2.75 ± 1.29	−2.85 ± 2.29	ns	−2.93 ± 2.08	−2.64 ± 1.31	−2.63 ± 1.36	ns
Z score SD L1–L4	−1.10 ± 1.06	−1.0 ± 1.13	−1.28 ± 1.08	ns	−1.13 ± 1.16	−1.1 ± 1.09	−1.26 ± 1.10	ns	−1.32^b^ ± 1.05	−0.98^a^ ± 1.12	−1.04^a,b^ ± 1.14	0.038
Hip BMD (g/cm^2^)	0.694 ± 0.09	0.700 ± 0.09	0.688 ± 0.08	ns	0.690 ± 0.08	0.691 ± 0.09	0.747 ± 0.05	ns	0.688 ± 0.08	0.700 ± 0.09	0.694 ± 0.09	ns
Hip T score^*^	−2.47^a^ ± 0.71	−0.22^a^ ± 0.81	−2.37^a^ ± 0.70	< 0.001	−2.49 ± 0.69	−2.31 ± 0.79	−1.95 ± 0.44	ns	−2.35^a^ ± 0.71	−2.23^a^ ± 0.80	−2.47^a^ ± 0.71	<0.001
Hip Z score SD	−0.93 ± 0.68	−0.63 ± 0.75	−1.28 ± 0.89	ns	−0.92^b^ ± 0.63	−0.64^a,b^ ± 0.83	−0.23^a^ ± 0.40	0.008	−0.70 ± 0.89	−0.63 ± 0.75	−0.93 ± 0.68	ns

“±” All DEXA values are given as mean ± SD

* Values followed by the same letters do not differ significantly

**Table 3:** Fracture incidence in individuals with the BsmI, ApaI and* Taq*I—genotypes

Type of fracture	BsmI genotype			*p*	ApaI genotype			*p*	*Taq*I genotype			*p*
	BB (*n* = 82)	Bb (*n* = 225)	bb (*n* = 193)		AA (*n* = 107)	Aa (*n* = 295)	aa (*n* = 135)		TT (*n* = 199)	Tt (*n* = 218)	tt (*n* = 84)	
Vertebral fractures (*n* = 167)	27	80	60	ns	41	83	44	ns	62	79	27	ns
No vertebral fractures (*n* = 333)	55	145	133		66	176	91		137	139	57	
Non-vertebral fractures (*n* = 117)	13	51	53	ns	18	59	40	ns	55	49	13	ns
No non-vertebral fractures (*n* = 338)	69	174	140		89	200	195		144	169	71	
All fractures (*n* = 284)	40	131	113	ns	59	142	84	ns	117	128	40	ns
No fractures (*n* = 216)	42	94	80		48	117	51		82	90	44	

*ns* non significant

Univariate tests showed correlations between T score and Z score at both hip and L1-L4 and selected genotype carriers (Table 3). However, none of these associations proved to be statistically significant in post hoc tests.

There were also no significant differences in fracture occurrence between individuals with the different genotypes of any studied VDR polymorphism.

## VDR single alleles

Table 4 shows the association analysis of single alleles of VDR polymorphisms with fracture incidence and BMD. The differences were detected for presence or lack of non-vertebral fractures. In the non-vertebral fracture group there were 77 single B alleles and 157-b, whereas in the group with no non-vertebral fractures the number of single B and b alleles was 312 and 454, respectively. Based on the presented frequencies of single alleles of the *Bsm*I VDR, B:b ratio was calculated as 1:2 and 1:1.5 for the fracture and no fracture groups, respectively (*p* = 0.032).

**Table 4:** Association analysis of BsmI, ApaI,* Taq*I single alleles of VDR with fracture incidence

	BsmI (*n* = 1000)				ApaI (*n* = 1,002)				*Taq*I (*n* = 1,002)			
	*n*	B (*n* = 389)	b (*n* = 611)	*p*	*n*	A (*n* = 473)	a (*n* = 529)	*p*	*n*	T (*n* = 616)	t (*n* = 386)	*p*
Vertebral fractures	334	134	200	ns	336	165	171	ns	336	203	133	ns
No vertebral fractures	666	255	411		666	308	358		666	413	253	
Non-vertebral fractures	234	77	157	0.032	234	95	139	0.021	234	159	75	0.020
No non-vertebral fractures	766	312	454		768	378	390		768	457	311	
All fractures	568	211	357	ns	570	260	310	ns	570	362	208	ns
No fractures	432	178	254		432	213	219		432	254	178	

Similarly, for T*aq*I polymorphism in the non-vertebral fracture group, the number of single T alleles was 159, and t alleles −75. In the no fracture group, there were 457 and 311 single T and t alleles, respectively. Thus, *T:t* ratio was 2:1 and 1.5:1 for the patients with previous non-vertebral fractures and for the no fracture group, respectively (*p* = 0.020).

## Discussion

Genetic association studies in osteoporosis often bring discrepant results. Our study found no direct association between fracture prevalence and specific genotypes of the analyzed VDR polymorphisms. Our results are consistent with previous studies, i.e. meta-analyses by Uitterlinden [12] and Fang [13], where no relationship between *BsmI, Apa*I, *TaqI* i *Foq*I polymorphisms with fracture risk was found. The results previously published by our team in a similar group of postmenopausal women are also in line with the current findings [10, 14].

Association analysis of single alleles made it possible to increase the sensitivity of the tests and revealed that the single alleles b, a, T of *BsmI, Apa*I, *TaqI* polymorphisms, respectively, were overrepresented in patients with non-vertebral fractures compared to B, A, t alleles. The same was not shown for vertebral fractures. The B allele was therefore—contrary to some previous data [15]—not shown to be protective for bone. Confirming the correlation between higher prevalence of the b allele and higher fracture risk, we made similar observations to those of Uitterlinden et al. [16].

Some clinical studies proved a correlation between VDR polymorphisms and incidence of low-energy fractures. A recent large meta-analysis by Ji et al., including over 6,600 subjects, revealed a modest but significant association between hip fracture prevalence and lower frequency of bb genotype [17]. Feskanich et al., in a group of women over 75 year of age, proved the BB genotype to be associated with a more than twofold increased risk of hip fracture compared with the bb genotype [18]. Their findings are also consistent with the results obtained by Garnero et al. [19].

Discordant results may be due to different populations studied/due to racial differences in BDM and fracture risk. Susceptibility to osteoporosis and fractures has been shown to be substantially lower in black subjects when compared to those of Caucasian and Asian origin [20, 21]. Studies by Morrison [9] and Nguyen [22] were carried out among ethnically diverse Australian population, additionally exposed to higher doses of UVB radiation, with potentially higher amounts of active vitamin D. In our study, the studied females were Caucasian.

It is more than likely that the VDR gene expression is affected by environmental factors. Several authors suggested that calcium homeostasis may play a role in this process. Stathopoulou et al. [23], showed that, under lower calcium intake (<680 mg/d), the presence of the B allele of *Bsm*I polymorphism and of the t allele of *Taq*I polymorphism increased the risk of osteoporosis by 118 and 132 %, respectively. In the group of the higher (>680 mg/d) calcium intake, the influence of the VDR alleles on BMD was insignificant. Thus, the authors concluded that adequate calcium intake “masked” the VDR genetic influence on the bone. On the other hand, Gennari et al. [24], observed that intestinal calcium absorption (self-assessment questionnaire) in healthy postmenopausal Italian women was significantly lower in BB and tt genotypes than in bb and TT genotypes, respectively, and in AABBtt genotype than in either aabbTT or AaBbTt genotypes.

Recently, as we gain new insights into vitamin D actions, interest in its non-genomic signaling pathways has increased, particularly regarding the trans-membrane calcium transport [25]. There is growing evidence that local vitamin D hydroxylation differs and depends on calcium availability. An experimental study by Anderson et al., [26] showed that with high dietary calcium, the activity of 1-α hydroxylase (CYP27B1) in the kidney is reduced, whereas in the bone it increases, promoting calcium incorporation into the bone. A negative correlation between serum vitamin D and mRNA CYP27B1 levels in the kidney, and positive—in the bone, was also noted. In other words, under the influence of high serum vitamin D, its hydroxylation in the bone is also more efficient, which results in adequate calcium availability in the bone. These findings further support the hypothesis that environmental factors, especially dietary calcium, may modulate the genotype-phenotype relationship, and therefore have an impact on the obtained results.

Secondly, it should be remembered the *BsmI, Apa*I, and *TaqI* polymorphisms do not have an effect on the final protein product, as they are found in the non-coding region of the VDR gene [27]. This fact highlights the importance of understanding the mechanisms by which these polymorphisms affect the VDR action.

Thirdly, the activities of other polymorphisms within the large VDR gene should not be underestimated. According to the NCBI database, 180 single nucleotide polymorphisms (SNPs) and seven haplotypes [27] have been found so far. It is therefore likely that the identification of specific polymorphisms may fail to provide definitive knowledge about the risk of fracture, and serve only as its approximation. In a recently published work, Ramagopalan and co-workers were able to find 2776 DNA-binding sites in the VDR gene by chromatin immunoprecipitation sequencing (Chip-seq) [28], indirectly proving the pleiotropic effects of vitamin D. The binding sites were located in the promoter region of the gene, which implies that even the smallest mutations might affect the VDR gene function.

We did not find any association of the studied VDR gene polymorphisms with BMD. Morrison initially reported lower BMD in BB and tt homozygotes of BsmI i* Taq*I polymorphisms [10]. Also, meta-analysis by Thakkinstian and co-workers [29], revealed weak but statistically significant association between B allele and lower BMD in the lumbar spine. Both mentioned polymorphisms are found in the same haplotype block of the VDR gene and are linked to each other. A paper by Ralston et al. [30], who also did not show such relationship, is more in line with our work.

In addition to that complex scenario, there are non-BMD-related risk factors for fracture, e.g. falls. Again, they are associated with vitamin D. Proper vitamin D status has been shown to improve muscle mass and strength and to have a beneficial effect on motor coordination. Numerous (ca. 400) risk factors for falls (i.e. old age, poor vision, impaired hearing, imbalance, neurological disorders, medications, architectural barriers at home, etc.) have been identified [31], but to consider of all them in a comprehensive assessment of risk fracture seems difficult, if not impossible. It is therefore assumed that the non-BMD fracture risk factors might have also been the reason of divergent results of the abovementioned studies.

Our results support the concept that population variants of the given genotype might be diverse and multifactorial. At the same time, we showed that there is no universal insight into the genotype-phenotype relation. Thus, each study that offers a possible explanation of this complex issue would be beneficial to clinicians.

The prevalence of the BB genotype in the studied population was 16.4 %, which is similar to the prevalence observed in other samples of Caucasians populations [32, 33], proving that our sample was representative. Since we investigated women with a specific disease—that is with a specific phenotype—the results seem to be no less valuable than the results of population studies. Another advantage is the large sample size and ethnic homogeneity.

In the multifactorial etiology of osteoporosis, the role of genetic factors is undeniable, but complex. We managed to show that the presence of the single alleles a, b and T of *ApaI, BsmI* and *TaqI* polymorphisms of the VDR gene may be a predictor of low-energy fractures. However, lack of association between the VDR gene polymorphisms and BMD suggests different ways in which the former contributes to fracture risk. The question whether and how VDR genotypes influence bone microarchitecture requires further investigation.
